# Naïve CD8^+^ T cell derived tumor-specific cytotoxic effectors as a potential remedy for overcoming TGF-β immunosuppression in the tumor microenvironment

**DOI:** 10.1038/srep28208

**Published:** 2016-06-16

**Authors:** Hong Hanh Nguyen, Therasa Kim, Sang Yun Song, Somang Park, Hyang Hee Cho, Sung-Hoon Jung, Jae-Sook Ahn, Hyeoung-Joon Kim, Je-Jung Lee, Hee-Ok Kim, Jae-Ho Cho, Deok-Hwan Yang

**Affiliations:** 1 Research Center for Cancer Immunotherapy, Chonnam National University Hwasun Hospital, Hwasun, Jeollanam-do, Republic of Korea; 2 Department of Medical Sciences, Chonnam National University Hwasun Hospital, Hwasun, Jeollanam-do, Republic of Korea; 3 Department of Thoracic and Cardiovascular Surgery, Chonnam National University Hwasun Hospital, Hwasun, Jeollanam-do, Republic of Korea; 4 Department of Hematology-Oncology, Chonnam National University Hwasun Hospital, Hwasun, Jeollanam-do, Republic of Korea; 5 Academy of Immunology & Microbiology (AIM), Institute for Basic Science (IBS), Pohang, Republic of Korea

## Abstract

Despite of the potential implications for cancer immunotherapy, conventional approaches using *in vitro* expanded CD8^+^ T cells have suboptimal outcomes, mostly due to loss of functionality from cellular exhaustion. We therefore investigated the phenotypic and functional differences among *in vitro* activated CD8^+^ T cells of three different sources, namely naïve (NT_eff_), memory (MT_eff_) and tumor-infiltrating lymphocytes (TIL_eff_) from human and mice, to better understand mechanisms behind potent effector functions and potential for overcoming current limitations. In line with the greater proliferation activity and longer telomere lengths of NT_eff_ populations, cells of naïve origin exhibited significantly less amounts of T cell exhaustion markers than those of MT_eff_ and TIL_eff_, and moreover, acquired distinct expression patterns of memory-promoting transcription factors, T-bet and Eomes, induced in a rapid and sustainable manner. NT_eff_ cells appeared to have lower expression of Foxp1 and were refractory to apoptosis upon TGF-β conditioning, implying better survival potential and resistance to tumor-induced immune suppression. Of CD8^+^ T cell pools activated to tumor-specific CTLs, naïve cell generated effectors possessed the most potent cytotoxic activity, validating implications for use in rational design of adoptive immunotherapy.

Adoptive immunotherapy, or the infusion of ex vivo activated and expanded tumor-specific CD8^+^ T cells into cancer patients, is a strategy involving removal of CD8^+^ T cells from the tumor environment and provision of stimulatory conditions necessary for their optimal activation, in attempts to overcome poor T-cell responsiveness to tumors. Adoptive T-cell transfer therapy was first attempted in the late 1980s to early 1990s, following the identification of the first tumor associated antigens and isolation of tumor reactive CD8^+^ T-cell clones from cancer patients. A sufficient number of activated CD8^+^ effector T cells were obtained and subsequently transferred intravenously into patients, mediating tumor elimination^1^. However, current articles have reported that immunotherapy employing the use of CD8^+^ cytotoxic T lymphocytes (CTLs) is limited by chronic activation and functional impairment of effector cells induced by immunosuppressive factors^2^,^3^,^4^. Investigation of these cells has revealed a so called ‘exhaustion profile’ that includes cell dysfunction, loss of effector function, and progressive increase in the amount and diversity of check point inhibitors such as programmed cell death protein 1 (PD1), cytotoxic T lymphocyte antigen 4 (CTLA4), lymphocyte activation gene 3 protein (LAG3), and killer cell lectin-like receptor G1 (KLRG1)^2^,^3^,^4^. It has also been shown that CTL function is altered by transforming growth factor-β (TGF-β), a lymphocyte inhibitor frequently overexpressed in the tumor mircroenvironment (TME) of multiple tumors^5^,^6^. Stephen *et al.*^7^ have characterized the mechanism of T cell unresponsiveness in TME driven by Forkhead box protein P1 (Foxp1) upregulation in response to TGF-β, by which antigen-primed CD8^+^ T cells are prevented from proliferating and promoting cytotoxic function.

The pool of lymphocytes used for adoptive immunotherapy can be derived from any of the CD8^+^ subsets, including tumor-infiltrating lymphocytes (TILs), naive T cells (T_N_), and antigen-experienced memory T cells (T_M_), which can be divided into central memory (T_CM_) and effector memory (T_EM_) subsets that differ in phenotype, homing, and function^8^. Memory CD8^+^ T-cell subsets are studied more often than naïve cells, because of their tumor specificity and greater ability to rapidly proliferate when exposed to previously encountered antigens. Moreover, Berger *et al.*^9^ showed that effector cells derived from T_CM_ rather than T_EM_ possess increased potential to survive and establish immunologic memory after infusion in macaques. These findings have been consistent in mice memory subsets^10^. However, a more recent study has suggested that T_N_ subsets may serve as a suitable candidate for immunotherapy because of their low level of ‘exhaustion’ in effector cells, sustained replicative potential correlative to telomere length, and minimal effector differentiation translating to greater efficacy after infusion^11^. Although self or tumor-reactive naïve CD8^+^ T cells have the capacity to respond to tumor antigens and differentiate into cytotoxic effector cells, some major limitations are the relatively low number of self/tumor-specific lymphocytes that result from negative selection during thymic maturation and inadequate magnitude of stimulation from antigens in the tumor environment^12^. Studies have shown that CD8^+^ T cells can be expanded by T cell growth factor interleukin-2 (IL-2) in the absence of T-cell receptor (TCR) signals, and in this situation, IL-2 can induce a unique signaling pathway that is associated with strong lymphocyte-specific protein tyrosine kinase/JAK3-dependent activation of the PI3K/AKT pathway^13^,^14^. Such signaling pathways not only induce rapid proliferation of IL-2 activated naïve CD8^+^ T cells but also upregulate expression of eomesodermin (Eomes), signifying the differentiation fate of primary naïve effectors into long-lived memory cells. Several current studies performed in murine models have demonstrated the role of T-box transcription factors, T-bet and Eomes, in regulation of both effector and memory functions where high expression of T-bet promotes effector function and Eomes is linked to formation of the long-term memory population^15^,^16^,^17^.

Considering some of the current limitations in adoptive T-cell transfer, we sought to evaluate the phenotypic and functional differences of effectors derived from naive, memory, and tumor-infiltrating CD8^+^ T cells (NT_eff_, MT_eff_, and TIL_eff_, respectively), and to further investigate the possibility of generating optimal tumor-reactive CTLs with high-dose IL-2 activation for application in clinical trials. We report that exhaustion phenotypes and expression kinetics of T-box transcription factors for NT_eff_ cell populations show possible advantages over other subgroups for use in cell transfer therapy. Our results also suggest that effector cells of naïve origin are relatively resistant to immunosuppressive effects of TGF-β with a trend in lower Foxp1 expression. High-dose IL-2 priming of naïve T cells successfully expands tumor-specific effector cells that exert favorable cytotoxic function, and serves as a potential means for overcoming some of the current barriers in immunotherapy.

## Results

### Successful expansion of human naïve, memory, and TIL CD8^+^ T cells *in vitro*

**Figure 1:**
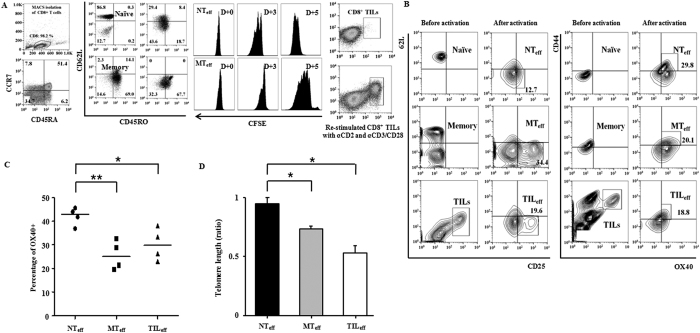
Characteristics of human naïve, memory, and TIL effector CD8^+^ T cells. (**A**) Isolation of CD8^+^ T cell subsets and proliferation assays. MACS isolation and FACS Aria analyses displayed *in vitro* expansion of naïve (CCR7^+^CD45RA^+^), memory (CD45RA^−^), and TIL (CD44^+^) subpopulations, 5 days post TCR stimulation. CFSE assay showed positive proliferation results after 3 to 5 days of culture. (**B**) Activation markers of effector cell subsets. Effector naïve and memory CD8^+^ cells (NT_eff_ and MT_eff_, respectively) are characterized by CD62L^-^CD25^+^CD44^+^OX40^+^ expression. Re-stimulated CD8^+^ TIL (TIL_eff_) populations also showed similar phenotypes. (**C**) OX40^+^ cell percentage of effector cell subsets. NT_eff_ showed significantly higher values compared to MT_eff_ and TIL_eff_ (*P < 0.05 vs TIL_eff_, **P < 0.005 vs MT_eff_). (**D**) Telomere length comparison showed the longest telomeres in NT_eff_ and shortest in TIL_eff_ cells (*P < 0.05). RTLs of effector cells were measured against CCRF-CEM control cells (1:1 ratio, total 5.0 × 10^5^ cells) with a DAKO Telomere PNA Kit. Data are representative of three to four independent experiments and are presented as mean ± SD.

CD8^+^ T cell subsets could be defined by the surface markers CD62L, CD45RO, CCR7, and CD45RA to naïve (CCR7^+^CD45RA^+^ or CD62L^+^CD45RO^−^) and memory (CD45RA^−^ or CD45RO^+^) cells. Tumor-infiltrating CD8^+^ T cells were CD44^+^, mostly with loss of homing receptors CD62L and CCR7 expression, and composed of two main sub-populations CD45RA^−^ and CD45RA^+^, suggesting a relatively differentiated status. After MACS isolation and stimulation by CD3/CD28 Dynabeads on anti-CD2 coated plates, successful expansion was demonstrated in all three groups by either CFSE peak division of NT_eff_ and MT_eff_ subsets, or enlarged populations of CD44^+^CD8^+^ TIL_eff_ cells (Fig. 1A). CFSE staining of TIL_eff_ proliferation was observed but peak division was indistinct due to the small number of initial TILs (data not shown). Activation kinetics differed for cell subsets where exponential expansion and accumulation was observed starting at day 3 for NT_eff_ and later at day 5 for MT_eff_ and TIL_eff_ cells, and thus further analyses of all effector cells were performed on day 5 post-stimulation.

Phenotypic characteristics of effector cell populations were assessed by surface expression of activation markers CD62L, CD25, CD44, and OX40. Effector cells from all progenitors (naïve, memory, and TILs) exhibited an effector phenotype with significant up-regulation of activation markers in comparison to their progenitors, and were considered activated as CD62L^−^CD25^+^CD44^+^OX40^+^ populations (Fig. 1B). Despite variability in the percentage of effector cells produced among donors, a significantly higher percentage of OX40 expressing cells was observed for NT_eff_ in comparison to MT_eff_ (p < 0.05) or TIL_eff_ (p < 0.005) cells (Fig. 1C). To further compare the proliferative potential among cell populations, relative telomere length (RTL), which correlate with replicative capacity, of NT_eff_, MT_eff_, and TIL_eff_ cells were investigated against CCRF-CEM control cell line. Telomere length was greatest in NT_eff_, shorter in MT_eff_, and shortest in TIL_eff_ cell subsets (Fig. 1D). This result was consistent with previously published reports of longer telomeres in human naive T cells, leading to increased proliferation of NT_eff_ cells in comparison to MT_eff_ cells after *in vitro* stimulation^9^.

### Exhaustion phenotypes differ among *in vitro* generated human and murine effector cells NT_eff_, MT_eff_, and TIL_eff_

**Figure 2:**
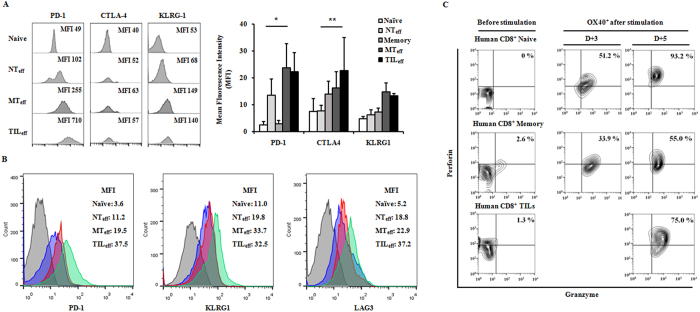
Exhaustion phenotypes of effector CD8^+^ T cell subpopulations in human and mice. Effector cells (OX40^+^) derived from naïve cells (NT_eff_) expressed lower levels of exhaustion markers PD1, CTLA4, KLRG1, and LAG3 compared to effectors from memory cells or TILs (MT_eff_ or TIL_eff_) (**A**) in human (*P < 0.05 for PD-1, **P < 0.005 for CTLA4) and (**B**) in mice at 5 days post-stimulation with anti-CD3/CD28 and anti-CD2. MFI levels are the average result of three experiments and are presented as mean ± SD. (**C**) *In vitro* functional analysis of human effector cells by cytokine production. An equal number of progenitors (2 × 10^5^ cells per subtype) activated to OX40^+^ effectors were characterized by flow cytometry for direct comparison among subtypes. NT_eff_ cells demonstrated a greater increase in perforin^+^ granzyme B^+^ populations compared to MT_eff_ and TIL_eff_ subsets at two time points (day 3 and 5 post-stimulation). Data are representative of three independent experiments.

Inhibitory receptors on T cell surfaces such as PD-1, CTLA-4, and KLRG-1, have been shown to facilitate T cell exhaustion by interaction with ligands on antigen presenting cells or tumor cells^3^,^4^,^5^. We therefore compared the expression of these inhibitory receptors on the three effector cell subtypes. All effector cells showed significant increase of exhaustion phenotypes during proliferation, but NT_eff_ cells showed significantly less expression of PD-1 and CTLA4 compared to MT_eff_ and TIL_eff_ subsets (Fig. 2A). Check point inhibitors showed varying levels of expression dependent on the time elapsed from activation with peak expression on days 4–5 for PD-1, days 5–7 for CTLA-4, and days 4–7 for KLRG-1 (data not shown). To investigate functional relevance of exhaustion phenotypes, we then evaluated the secretory function of cytotoxic cytokines such as granzyme B, perforin, and IFN- ɤ from different human effectors. During days 3 to 5 post-stimulation, the secretion of granzyme B and perforin gradually increased in all effector cells derived from naïve, memory, and TIL populations (Fig. 2C). Among fully activated day 5 effector cells, NT_eff_ possessed the highest expression level of perforin and granzyme B, and similar levels of IFN-ɤ (data not shown) compared to MT_eff_ and TIL_eff_ cells. In the murine model, effectors derived from CD8^+^ naïve and memory phenotype (MP) T cells, and TILs collected from OVAp-expressing EL4-EG7 tumors were successfully proliferated with CD44^+^OX40^+^ phenotypes. Expression levels of inhibitory receptors PD-1, KLRG1, and LAG3 in murine CD8^+^ effectors showed similar patterns to those of human cells (Fig. 2B).

### Expression kinetics of transcription factors T-bet and Eomes differ among CD8^+^ T cell effector populations

**Figure 3:**
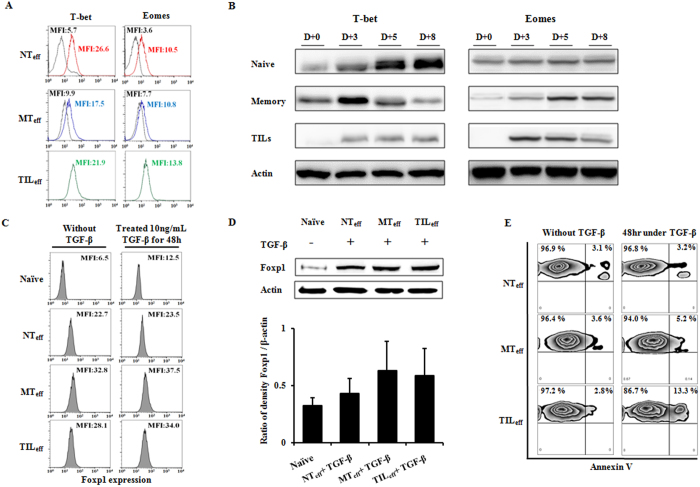
Expression of T-box transcriptional factors and characteristics of human effector CD8^+^ T cells under TGF-β conditioning. (**A**) Intracellular staining of T-bet and Eomes expressed on TCR stimulated effectors NT_eff_, MT_eff_, and TIL_eff_. MFI levels are shown for day 0 (initial peak) and day 5 (right side peak) post-stimulation. For TILs, day 0 peaks are lost due to insufficient cell amount pre-expansion. (**B**) Western blot analysis of naïve and memory T cells, and TILs on days 0, 3, 5 and 8 post TCR activation. Kinetics of T-bet and Eomes expression in differentiating CD8^+^ T cells suggest rapid and sustained stimulation for NT_eff_ populations. Effector groups from day 5 post-stimulation are treated with TGF-β (10 ng/mL) for 48 hours and checked for Foxp1 expression (day 7 post-stimulation) by (**C**) intracellular FACS staining and (**D**) Western blot. Increase in Foxp1 MFI levels pre and post TGF-β conditioning was minimal for naïve effector cells and NT_eff_ showed the least Foxp1 protein expression, although not statistically significant. MFI levels and protein density ratios are the average result of three experiments and shown as mean ± SD. (**E**) Annexin V assay by flow cytometry. Quadrant plots show the percent distribution of early and late apoptotic cells (first quadrant) with or without 48-hour exposure to TGF-β. Naïve cell derived effectors (NT_eff_) showed minimal increase in apoptotic cell populations relative to MT_eff_ and TIL_eff_ subgroups. All experiments were repeated three times and are presented by one representative figure.

For further characterization of the CD8^+^ T cell subpopulations, the expression levels of two T-box transcription factors, T-bet and Eomes, were examined using intracellular staining for FACS analysis and confirmed with Western blot. Although naïve CD8^+^ T cells expressed lower levels of T-bet and Eomes in comparison to memory cells before stimulation, activated effectors NT_eff_, MT_eff_, and TIL_eff_ showed relatively similar MFI levels of T-bet and Eomes, demonstrating the capacity of naïve or memory cells to induce similar effector populations (Fig. 3A). However, considering the lower MFI for pre-stimulated naïve cells, this cell population displays a greater increase in T-bet and Eomes expression levels post-stimulation compared to memory T cells.

To clarify and confirm the expression profile of these T-box transcription factors in the effector subpopulations, Western blot analysis was performed using whole cell lysates of naïve and memory T cells, and TILs on days 0, 3, 5 and 8 post TCR activation. Our experiments revealed clear differences in the kinetics of T-bet and Eomes expression during CD8^+^ T cell activation (Fig. 3B). Effectors derived from naïve cells increased in T-bet expression appreciably starting on day 3, reaching maximum on day 8, whereas memory cell derived effectors gained the highest expression of T-bet earlier on day 3 and were quickly down-regulated on days 5 through 8. TIL_eff_ cells demonstrated poor expression of T-bet throughout stimulation time. While naïve and memory cells showed increasing Eomes expression with stimulation, TILs exhibited decreasing intensity during day 5 through 8 post-activation. Such kinetics suggest that NT_eff_ cells express higher levels of both T-bet and Eomes, and expression is sustained for longer periods during activation, in comparison to other effector groups MT_eff_ and TIL_eff_. Collectively, naïve T cell derived effectors appear to differentiate relatively rapidly, proliferate actively, and have potential to be converted into long-lived memory cells.

### Foxp1 expression and apoptosis studies under TGF-β conditioning show favorable results for CD8^+^ NT_eff_ cells

To determine the immunosuppressive resistance potential of CD8^+^ NT_eff_ cells in the tumor microenvironment, we examined expression of Foxp1 among effector cells derived from naïve, memory, and TIL progenitors in the presence of TGF-β. After 5 days of proliferation, effector cells were exposed to TGF-β (10 ng/mL) for 48 hours and Foxp1 expression (day 7 post-stimulation) was evaluated by intracellular FACS staining. MT_eff_ and TIL_eff_ populations showed increased Foxp1 expression with TGF-β conditioning, whereas NT_eff_ cells maintained their lower expression levels in three independent experiments (Fig. 3C). To confirm the increased expression of Foxp1 in CD8^+^ effector cells in the tumor microenvironment, Western blot analysis was performed for effector T cell subsets under TGF-β exposure using non-stimulated naïve cells as the control. As expected, NT_eff_ populations expressed the lowed amount of Foxp1 protein expression relative to other effector groups, consistent with intracellular staining results (Fig. 3D). After conditioning with TGF-β, flow cytometry detection of apoptosis with Annexin V displayed limited induction of apoptotic cell populations for NT_eff_ cells relative to MT_eff_ and TIL_eff_ subgroups (Fig. 3E). Taken together, these results suggest that CD8^+^ effector subgroups differ in Foxp1 expression and apoptotic response to TGF-β exposure, and that NT_eff_ cells maintain resistance to inhibitory factors induced from the tumor microenvironment.

### Cytotoxic activity against targeted tumor cells is superior for effector cells of naïve T cell origin in both human and mouse models

**Figure 4:**
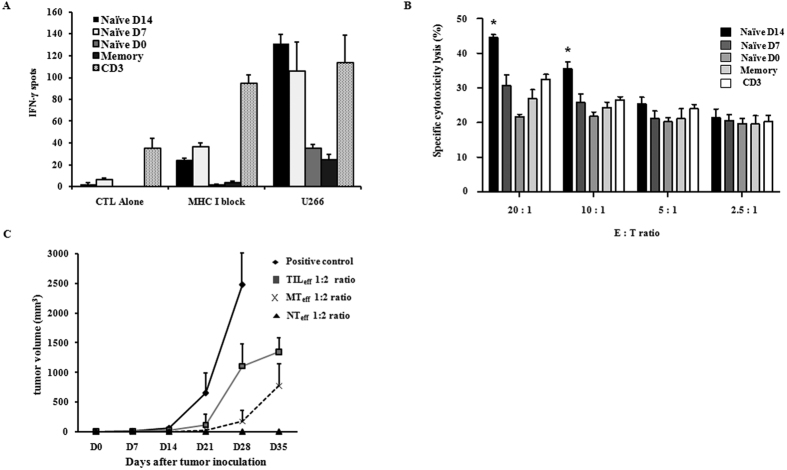
Tumor-specific CTL function of *in vitro* human and *in vivo* murine CD8^+^ T cells. (**A**) *In vitro* comparison of tumor-specific CTL function of IL-2 primed human CD8^+^ T cells against U266 MM cells by IFN-ɤ ELISPOT assay. CTL alone represents the control group and U266 refers to cytotoxic activity of effectors against U266 target cells (E:T ratio of 10:1) in the absence of MHC class I restriction, expressed as the number of IFN-ɤ spots observed. Naïve T cells activated under high-dose IL-2 for 14 days (Naïve D14) displayed highest activity against U266 target cells. (**B)** LDH cytotoxicity assay of IL-2 primed human CD8^+^ T cells. Specific cytotoxicity lysis percentages against U266 MM target cells were highest for Naïve D14 effectors compared to other groups when E:T ratios were at least 10:1 (*p < 0.05). (**C**) *In vivo* CTL activity in the murine model. Following injection of NT_eff_, MT_eff_, and TIL_eff_ CD8^+^ T cells, OVAp-expressing EL4-EG7 cells (8 × 10^5 ^cells/200 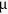
L) were inoculated subcutaneously in B6 recipient mice in a 1:2 ratio of target tumor to effector cells. All B6 mice injected with TIL_eff_ had positive mass (1 cm^3^) formation after 28 days, whereas tumor growth was suppressed in NT_eff_ harboring B6 mice for longer than 35 days. All data are representative of three independent experiments and shown as mean ± SD.

To investigate the cytotoxic function of tumor-specific human CTLs, antigen (U266 MM cell line) loaded autologous mature dendritic cells (mDCs) were co-cultured for 7 days with IL-2 primed naïve and memory CD8^+^ T cells and the generated CTLs were assessed via IFN-ɤ ELISPOT assay and LDH Cytotoxicity Detection Kit. A total of five cell groups were harvested for cytotoxic analyses including CD8^+^ (Naïve D14, Naïve D7, Naïve D0, and Memory) and CD3^+^ CTLs. Briefly, Naïve D14 and Naïve D7 cells refer to CD8^+^ naïve cells co-cultured with high-dose IL-2 (1 μg/mL) for 14 and 7 days respectively, whereas Naïve D0 and Memory cells are co-cultured with conventional dose IL-2 (10 ng/mL) and are of naïve and memory T cell origin, respectively (refer to Methods). IFN-ɤ ELISPOT was measured in the presence and absence of MHC class I specific monoclonal antibody (W6/32, 20 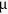
g/mL) with CTL alone as the control. High dose IL-2 primed Naïve D14 effector cells showed the highest secretion of IFN-ɤ against target U266 cells when co-cultured for one day at a 10:1 ratio of effector (E) to target (T) cells (Fig. 4A.). Although CD3^+^ cells also secreted higher levels of IFN-ɤ relative to Naïve D7, Naïve D0, and Memory CD8^+^ cells, they also displayed non-specific secretion as observed in the control CTL alone group. Direct cytotoxicity testing against U266 target cells using the LDH cytotoxic assay revealed enhanced CTL activity for Naïve D14 cells that was significantly greater than other effector CTLs when E:T ratios were at least 10:1 (Fig. 4B.). In the murine model, *in vivo* CTL activity against inoculated OVAp-expressing EL4-EG7 lymphoma cells in B6 mice was examined for NT_eff_, MT_eff_, and TIL_eff_ CD8^+^ effector subsets obtained from OT-1 Thy1.1 mice. Tumor growth was negative for at least 35 days after tumor inoculation in mice injected with NT_eff_ cells, whereas MT_eff_, and TIL_eff_ injected mice showed positive tumor growth within the same time frame (Fig. 4C). Tumor growth became positive in one NT_eff_ injected mouse at 42 days post inoculation (data not shown).

## Discussion

Current adoptive immunotherapy involves mainly the use of CD3-collected T cells or autologous genetically engineered TILs, with increasing prospect for memory CD8^+^ cells^18^,^19^. Autologous CD8^+^ subpopulations hold the advantage of avoiding contamination of immunosuppressive regulatory T cells and decrease in terminal differentiation with repeated clonal proliferation in cancer patients. In order to demonstrate the superior background of tumor-specific CTLs derived from naïve CD8^+^ populations we focused on clarifying the different exhaustion characteristics, including expression check point inhibitors and transcription factors, and divergence of cytotoxic function among different effector groups NT_eff_, MT_eff_, and TIL_eff_. We found that naïve CD8^+^ effectors have relatively favorable resistance patterns to immunosuppression and strong CTL potency against tumor targets. This may be explained by the “fresh” nature of these cell groups that display lower levels of inhibitory immune checkpoint receptors such as PD-1, CTLA-4, and KLRG1, leading to a greater activation and replicative potential^20^. Our results were consistent with reports by Hinrichs *et al.*^21^ indicating effectors derived from naïve CD8^+^ T cells may be the preferable candidate for T-cell adoptive immunotherapy based on evaluation of KLRG1, CD57, CD27 expression and telomere length. Such markers represent tendency of MT_eff_ or TIL_eff_ cells to quickly enter into a T cell “exhaustion” phase with elevated levels of inhibitory receptors following long-term exposure to tumor antigen and ultimately acquire a non-functional state^4^,^22^,^23^.

Another point of consideration is the expression of two T-box transcriptional factors, T bet and Eomes, as well as their effect on CTL differentiation. Among various transcription factors that function in pairs to regulate effector and memory CD8^+^ T cell development, short- and long-term effects of T-bet and Eomes have been relatively well identified in the murine model, but are still limited to the context of infection and disease in humans^16^,^24^,^25^,^26^,^27^. Previous studies have emphasized on the role of high T-bet expression, which is associated with long-term resilience, low expression of inhibitory receptors, and protection from CD8^+^ T-cell exhaustion^27^,^28^, as well as Eomes induced inhibition of cell death leading to generation or survival of memory cells^17^,^29^. In our experiments, data showed that NT_eff_ cells gradually increase and maintain high expression levels of both T-bet and Eomes throughout activation, whereas other effectors quickly down-regulated these factors. It has been suggested that high expression of T-bet correlates with a granzyme B^+^perforin^+^ phenotype where high perforin secretion was only detected in T-bet^hi^Eomes^hi/lo^ cells^15^. Stable expression of Eomes levels in NT_eff_ and MT_eff_ subsets show their ability to become memory-precursor effector cells (MPECs), whereas TIL_eff_ cells enter a terminal differentiation phase via down regulation of Eomes. Collectively, our data indicates that NT_eff_ populations co-express sustained, high levels of T-beta and Eomes, and a granzyme B^+^perforin^+^ phenotype representative of full cytotoxic function.

To clarify the relationship between patterns of Foxp1 expression and TGF-β, we activated T cells by *in vitro* TCR stimulation and evaluated Foxp1 levels of these effectors before and after TGF-β exposure with intention to mimic the immunosuppressive TME. As shown in a recent article^7^, overexpression of Foxp1 is thought to be mediated by TGF-β signaling through Smad protein interaction, which in turn mediates c-Myc and c-Jun transcriptional repression, and is expected to be upregulated after homing to the TME. However, in this study, only whole mouse CD8^+^ cells were investigated and did not address subsets of human CD8^+^ T cells. As inferred from our results, CD8^+^ NT_eff_ are less influenced by tumor-derived inhibitory factor TGF-β, i.e., effectors derived from naïve cells are less affected by tumor suppression mechanisms driven by Foxp1 in response to TGF-β. Importantly, 48 hours post treatment of TGF-β, a smaller percentage of NT_eff_ cells entered apoptosis compared to MT_eff_ and TIL_eff_ subsets, which further offers evidence for the superior features of naïve cell derived effectors in resistance to the TGF-β induced apoptosis effect. These findings provide indication for the use of TGF-β inhibitors^29^, NT_eff_ engineered to express a dominant-negative mutant of the TGF-β receptor^30^, and Foxp1-deficient NT_eff_, with objectives to enhance the immunosuppressive resistance of lymphocytes in the TME in various clinical applications.

The essential role of high-dose IL-2 in cancer has now been widely discussed, especially because IL-2 is suspected to optimize all stages of CD8^+^ T cell response, including primary expansion, contraction, memory generation, and secondary expansion^31^. In addition, high-dose IL-2 may strongly induce the proliferation as well as stimulation of naïve CD8^+^ T cells both *in vitro* and *in vivo*^32^. In our research, high-dose IL-2 (1 μg/mL) stimulated naïve CD8^+^ T cells, which were then co-cultured with autologous DCs, could successfully generate strong CTLs within a short duration. By evaluation of ELISPOT and LDH assay, Naïve D14 and Naïve D7 both generated CTLs with a high number of IFN-ɤ spots and better (Naïve D14) or similar (Naïve D7) cytotoxic effects compared to CD3^+^ CTLs. As mentioned, high concentrations of IL-2 (0.1–1 μg/mL) are necessary for stimulation of CD8^+^ naïve T cells through the antigen-independent manner^13^. Although our antigen-loaded DCs could induce antigen-specific CTLs from CD8^+^ naive T cells, higher than conventional dose IL-2 was required not only for proliferation of the limited number of antigen-specific naïve CD8^+^ T cells but also for generation of potent CTLs. During cytokine stimulation, high-dose IL-2 plays an important role in both pre-activation (during 7 days co-culture with naïve T cells) and co-activation (during 7 days co-culture with IL-2 primed T cells and DCs). In the co-activation period, high-dose IL-2 could potentially be used with other ɤ_c_ cytokines (IL-7, IL-15) for greater generation of CTLs. However, the optimal exposure times of high-dose IL-2 to tumor-specific naïve CD8^+^ T cells remains to be determined.

Taken together, our results provide a relatively complete framework for collecting effector cells derived from naïve origin and present a practical method to effectively generate strongly functional NT_eff_ populations with the use of high-dose IL-2 (1 μg/mL). Nowadays, human T lymphocytes, which can be genetically engineered prior to adoptive transfer, can express virtually any target gene, by application of techniques involving genes encoding T-cell receptors (TCRs) or chimeric antigen receptors (CARs) to provide the desired T-cell specificity^11^. CAR-redirected T lymphocytes (CAR-T cells) are mostly limited to memory and effector T cells^33^,^34^,^35^ due to potent cytotoxic function. However, the appropriate substrate of effector T cells for clinical use is still controversial and the development of antigen escape variants may represent the limitation of monoclonal specificity. To obtain an adequate amount of effector cells with exceeding cytotoxic function, we suggest that naïve cells should be considered as a candidate for CAR-T therapy.

In summary, primary effector cells derived from naïve CD8^+^ T cells showed the highest potential for adoptive cell transfer therapy, with lower expression of inhibitory surface markers, higher secretion of cytotoxic cytokines, and improved resistance to TGF-β induced suppression in the TME, in comparison to secondary effectors derived from memory or TIL CD8^+^ cells. High-dose IL-2 priming amplifies the number of tumor-specific naïve CD8^+^ T cells without clonal exhaustion and results in the generation of potent tumor-reactive CTLs with capacity to overcome tumor-derived immune suppression.

## Methods

### Ethical approval of studies and informed consent

All experimental protocols were approved by the institutional ethical committee at ChonnamNational University Hwasun Hospital (CNUHH2014-146) and methods were carried out in accordance with the approved guidelines. Human peripheral blood and tumor masses were donated from healthy donors or lung cancer patients after obtaining written informed consent from all subjects. Animal experiments were performed with ethical approval from the Chonnam National University Animal Research Committee (HCRL15001-2).

### Isolation of human CD8^+^ T cell subsets and tumor-infiltrating lymphocytes (TILs)

Peripheral blood mononuclear cells (PBMCs) were obtained from healthy donors by density gradient centrifugation with Lymphoprep (AXIS-SHIED Rodelokka, Oslo, Norway). CD3^+^ and CD8^+^ lymphocytes were isolated by a magnetic-activated cell sorter (MACS) using CD3 Microbeads and a CD8 T cell isolation kit (MiltenyiBiotec), respectively. The cells were then labeled with anti-CD8, anti-CCR7, and anti-CD45RA (Ebiosciences) and sorted into naïve and memory CD8^+^ T cell subsets on a BD fluorescence activated cell sorting (FACS) Aria sorter (BD Biosciences). For extraction of TILs, tissue from lung tumor samples were minced and digested with collagenase (2.5 mg/ml collagenase I) at 37 °C for one hour. Cell suspension was then twice filtered through 100-μm and 40-μm cell strainers (BD Biosciences) to obtain single cells. TILs were isolated from single cells utilizing density gradient centrifugation with Lymphoprep and further purified using CD8 MicroBeads (MiltenyiBiotec).

### Generation of human CD8^+^ effector subgroups and proliferation assay

Isolated naïve, memory, and TIL subpopulations were mixed in a 1:1 ratio with Human T-Activator CD3/CD28 Dynabeads (Gibco, Life Technologies) and cultured on CD2-coated plates (CD2-Biotin functional grade, MiltenyiBiotec). Carboxyl fluorescein succinimidyl ester (CFSE) studies were performed by labeling cells with 2.5 μM CFSE (Life Technologies) before stimulation. Three and 5 days post-stimulation, CFSE division of cells was determined by flow cytometry and analyzed using FlowJo Version 10.0.7 software. After 5 days of stimulation, activation of effector cells was determined by FACS analysis based on CD62L, CD25, CD44, and OX40 expression.

### Telomere lengths

Relative telomere lengths (RTL) of effector cells were determined by comparing test cells to a control cell line (human T-cell lymphoblast-like cell line CCRF-CEM, Sigma-Aldrich) using a DAKO Telomere PNA Kit (DAKO). Test cells (NT_eff_, MT_eff_, and TIL_eff_) and control cells (cell line 1301) were washed in PBS and mixed in a 1:1 ratio. A total of 5 × 10^5^ cells were resuspended in 300 μl of hybridization solution containing 70% formamide either without a probe (unstained control) or with a fluorescein-conjugated telomere PNA probe. The cells were heated for 10 min at 82 °C for DNA denaturation and hybridization was performed overnight at room temperature. Subsequently, cells were washed and resuspended in 0.5 ml of DAKO DNA staining solution and incubated at 2–8 °C for 2 hours. Samples were then analyzed by flow cytometry using logarithmic scale FL1-H for probe fluorescence and linear scale FL3-H for DNA staining. RTLs of the sample cells were calculated per manufacturer’s instructions and reported as mean ± standard deviation (SD) for triplicate samples.

### Surface and intracellular staining using FACS

For surface staining, cells were labeled with mAbs against various targets including CD8, CD62L, CCR7, CD45RA, CD45RO, CD25, CD44, OX40, PD-1, CTLA-4, and KLRG-1 (Ebiosciences), incubated at 4 °C for 15 minutes, then washed twice with PBS 1% FBS (FACS buffer), and finally fixed in PBS containing 1% paraformaldehyde (Fix buffer). For T-bet (4B10) and Eomes (Dan11mag), and Foxp1 intracellular staining, cells were first labeled with surface markers CD8, CD62L, OX40 (Ebiosciences) for T-bet and Eomes, or CD25 (BD Biosciences), CD8, CD27, and CD44 (Ebiosciences) for Foxp1 detection, respectively, and then fixed and permeabilized with Foxp3/Transcription Factor Fixation/Permeabilization Concentrate and Diluent (Ebiosciences) in a 96-round-well plate. Intracellular labeling for T-bet and Eomes (Ebiosciences), and Foxp1 (LifeSpan Technologies) was performed according to manufacturer’s instruction. For evaluation of Perforin, Granzyme B, and IFN-ɤ cytokine secretion, effector cells were incubated with brefeldin A for 4 hours at 37 °C to disrupt Golgi-mediated transport and accumulate cytokines. Cells were then surface stained, permeabilized with BD Cytofix/Cytoperm™ Fixation/Permeabilization Solution Kit (BD Biosciences), and intracellularly stained as recommended. Flow cytometry data was analyzed using FlowJo Version 10.0.7 software.

### Apoptosis under TGF-β conditioning

For evaluation of apoptosis, individual effector CD8^+^ T cell populations were exposed under TGF-β (10 ng/mL) 48 hrs and stained with Annexin V FITC (FITC Annexin V Apoptosis Detection Kit I). Briefly, 1 × 10^6^ cells were washed twice with cold PBS and stained with 5 μL of Annexin-V-FITC in binding buffer for 15 minutes at room temperature. Apoptotic cells were evaluated by flow cytometry according to the manufacturer’s protocol (BD Pharmigen).

### Western blot protocol

For Western analysis, whole-cell protein lysates were obtained from effector CD8^+^ T cells at 4 time points (0, 3, 5, and 8 days post-stimulation) with lysis buffer Proprep (INTRON Biotechnology) by suspending 106 cells in 10 μl buffer and incubating on ice for 30 minutes in the presence of Halt Protease & Phosphatase inhibitors cocktail (Thermo Scientific). Cell lysates were resolved by 10% Bis-Tris SDS PAGE Gel, transferred onto PVDF membrane (Merck), blocked with 5% skim milk in TBST buffer containing 0.1% Tween-20, and probed with the following mAbs: T-bet (4B10, Santa Cruz), Eomes (Y-20, Santa Cruz), Foxp1 (polyclonal, Abcam), and β-actin (C4, Santa Cruz). Quantification of detected protein was performed with an Intelligent Dark Box unit (LAS-3000; Fujifilm) and normalized for loading with the amount of β-actin (1:5000) detected in each lane.

### Generation of human tumor-specific CTLs

Mature dendritic cells (mDCs) were pulsed with apoptotic body (irradiated U266 cell line) at a 2:1 ratio (2 × 10^5^ DC: 10^5^ U266) from day 6 to day 8 and then presented to CD8^+^ T cells at a 1:10 ratio (2 × 10^5^ DC: 2 × 10^6^ CD8^+^ T cell). Naïve and memory T cells were isolated from the same donor and cultured in different conditions. To visualize cytokine-activated proliferation and determine the optimal co-culturing start time with antigen-loaded mDCs post high-dose IL-2 activation, activated naïve CD8^+^ T cells were labeled with CFSE and analyzed at various time points (Supplementary Fig. S1). Initiation of peak expansion at 7 days post high-dose IL-2 lead to the decision to start co-culture of cells with mDCs after 7 days of high-dose IL-2 pretreatment. Naïve cells were activated with high-dose IL-2 (1 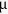
g/mL) in two conditions; 14 day exposure to high-dose IL-2 (Naïve D14; 7 days pretreatment and during 7 days of mDC co-culture) and 7 day exposure to high-dose IL-2 (Naïve D7; 7 days pretreatment only followed by 7 days of mDC co-culture without high-dose IL-2). For control, we cultured naïve and memory cells under equal conditions; 7 day mDC co-culture with IL-2 (10 ng/mL) and IL-7 (10 ng/mL), labeled Naïve D0 and Memory, respectively. After mDC co-culture, 4 groups of CD8^+^ CTLs (Naïve D14, Naïve D7, Naïve D0, and Memory) and CD3^+^ CTLs (cultured as previously described) were harvested for cytotoxic analysis.

### Cytotoxic functional evaluation of human tumor-specific CTLs

The U266 cell line was chosen as target cells for our cytotoxic assays based on its expression of E-Cadherin and PD-L1 (Supplementary Fig. S2), which are ligands of KLRG-1 and PD-1, respectively. ELISPOT assay (BD Biosience) was performed to quantify antigen-specific IFN-ɤ releasing effector T cells. 2 × 10^5^ of the effector T cells were co-cultured with 2 × 10^4^ U266 target cells in a 96-well nitrocellulose flat-bottom plate for 24 hours at 37 °C. IFN-ɤ ELISPOT was measured in the presence or absence of MHC class I specific monoclonal antibody (W6/32) with CTLs alone as the control, and spots were counted with ImmunoSpot Reader (Cellular Technology Ltd, Ohio). Data is presented as the mean number of spots ± SD of IFN- ɤ secreting cells per well of triplicate samples.

Evaluation of CD8^+^ and CD3^+^ CTL functional activity was performed using the Cytotoxicity Detection Kit LDH (Roche Applied Science, Basel, Switzerland) according to the manufacturer’s protocol^36^,^37^. Cytotoxicity of CTLs was calculated according to the following formula: % cell lysis = (experimental – effector spontaneous – low control) x 100/(high control – low control), where “experimental” corresponds to the experimental signal value, “effector spontaneous” to the spontaneous background signal value of the effector cells alone, “low control” to the spontaneous background signal value of target tumor cells alone, and “high control” to the maximum signal value of target cells in medium containing 1% Trixton X-100. Cytotoxicity assays were performed for varying effector cell (E) to target cell (T) ratios, and specific cytotoxicity lysis percentages are reported as the mean ± SD of triplicate samples.

### T cell purification, *in vitro* stimulation, and *in vivo* CTL assay of the murine model

C57BL/6 (B6) mice were purchased from Orient Bio (Iksan, South Korea) and OT-1.Thy1.1 TCR transgenic mice on a B6 background were obtained from the Institute for Basic Science (IBS; Pohang, South Korea). All mice were used in experiments at 6 to 12 weeks of age according to protocol. Pooled lymph nodes from OT-1 transgenic mice were stained with fluorochrome-conjugated antibodies to CD4, CD8, CD25, CD44 and CD62L and sorted to obtain CD44^−^CD62L^+^ naïve or CD44^+^ memory-phenotype (MP) CD8^+^ T cells using FACS. CD8^+^ TILs were obtained from B6 mice that were inoculated subcutaneously with OVAp-expressing EL4-EG7 tumor cell lines. For *in vitro* stimulation, sorted naïve, MP, and TIL CD8^+^ T cells were cultured on plate-bound anti-CD3 and anti-CD28 with IL-2 (10 ng/mL) and IL-15 (10 ng/mL). Effector cell surface staining procedure for PD-1, KLRG-1, and LAG3 labeling was identical to human cell methods previously described. *In vivo* CTL functional assay was performed using OVAp-expressing EL4-EG7 tumor cell lines as target cells. EL4-EG7 tumor cells (0.8 × 10^5^ cells/200 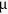
L) were inoculated subcutaneously in recipient B6 mice and 8 hours later, NT_eff_, MT_eff_, and TIL_eff_ were injected intravenously in a 2:1 ratio of effector to target cells. A tumor mass growth of greater than 1 cm^3^ was considered as positive tumor formation.

### Statistical analysis

The two-tailed student’s t test was used to determine differences in the mean. P values of less than 0.05 were considered statistically significant.

## Additional Information

**How to cite this article**: Nguyen, H. H. *et al.* Naïve CD8^+^ T cell derived tumor-specific cytotoxic effectors as a potential remedy for overcoming TGF-β immunosuppression in the tumor microenvironment. *Sci. Rep.* **6**, 28208; doi: 10.1038/srep28208 (2016).

## Supplementary Material

Supplementary Information
