# Supplementary material for: Naïve CD8+ T cell derived tumor-specific cytotoxic effectors as a potential remedy for overcoming TGF-β immunosuppression in the tumor microenvironment
Source: Sci Rep. 2016 Jun 16;6:28208. doi: 10.1038/srep28208 (PMC4910083; doi:10.1038/srep28208)

Naïve CD8+ T cell derived tumor-specific cytotoxic effectors as a potential remedy for overcoming TGF-β immunosuppression in the tumor microenvironment

Hong Hanh Nguyen[1], Therasa Kim[2], Sang Yun Song[3], Somang Park[1], Hyang Hee Cho[1], Sung-Hoon Jung[4], Jae-Sook Ahn[4], Hyeoung-Joon Kim[4], Je-Jung Lee[1,4], Hee-Ok Kim[5], Jae-Ho Cho[5] and Deok-Hwan Yang[1,4]

[1]Research Center for Cancer Immunotherapy, [2]Department of Medical Sciences, [3]Department of Thoracic and Cardiovascular Surgery, [4]Department of Hematology-Oncology, Chonnam National University Hwasun Hospital, Hwasun, Jeollanam-do, Republic of Korea; and [5]Academy of Immunology & Microbiology (AIM), Institute for Basic Science (IBS), Pohang, Republic of Korea

Supplementary Figure S1. Proliferation assay of high-dose IL-2 (1 μg/mL) primed human naïve $CD8^+$ T cells. CFSE analyses at various time points showed adequate peak expansion starting at 7 days post high-dose IL-2 stimulation.

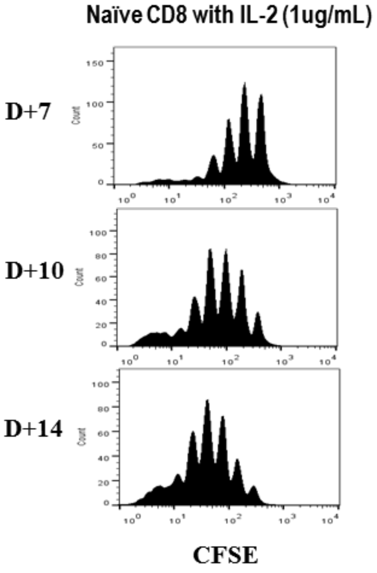

Supplementary Figure S2. Expression of PD-L1 and E-Cadherin in different tumor cell lines. The U266 cell line was chosen as target cells for cytotoxic assays based on its expression of E-Cadherin and PD-L1

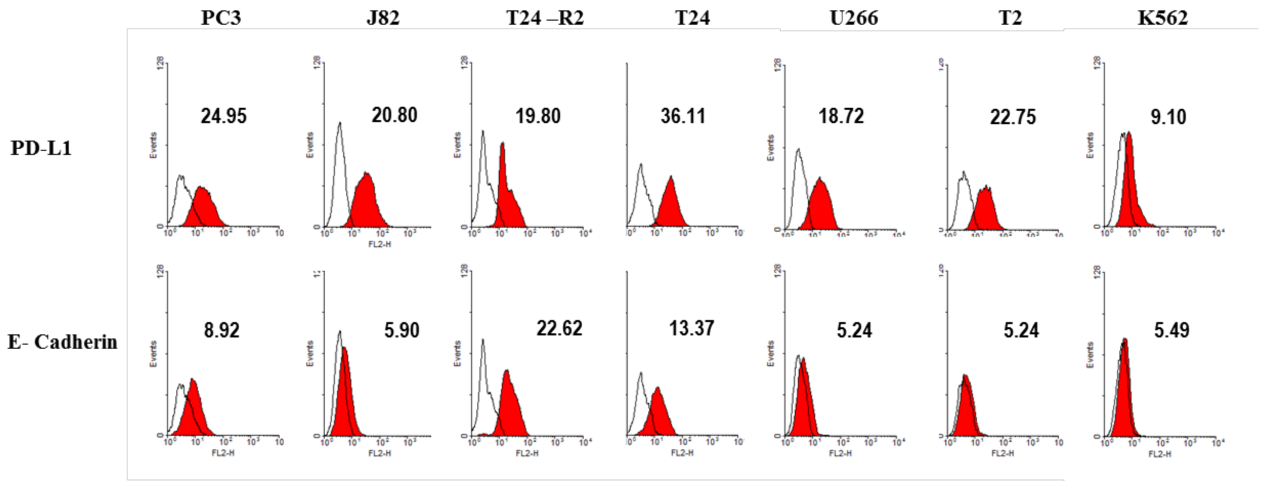

Supplement: Supplementary Information [file srep28208-s1.pdf]
